# Quercetin and AMPK: A Dynamic Duo in Alleviating MG-Induced Inflammation via the AMPK/SIRT1/NF-κB Pathway

**DOI:** 10.3390/molecules28217388

**Published:** 2023-11-02

**Authors:** Ziyin Lu, Haozhen Wang, Muhammad Ishfaq, Yufang Han, Xiujin Zhang, Xiang Li, Baoqi Wang, Xiuli Lu, Bing Gao

**Affiliations:** 1 School of Life Science, Liaoning University, Chongshanzhong-Lu No. 66, Shenyang 110036, China; ziyinlu@lnu.edu.cn (Z.L.); wanghzlnu@foxmail.com (H.W.); han746480@163.com (Y.H.); zhangxiujin1213@163.com (X.Z.); nadh990205@163.com (X.L.); wang1286849062@163.com (B.W.); 2 College of Computer Science, Huanggang Normal University, Huanggang 438000, China; muhammad@hgnu.edu.cn; 3 Department of Cell Biology and Genetics, Shenyang Medical College, Shenyang 110034, China

**Keywords:** *Mycoplasma gallisepticum*, quercetin, AMPK, inflammation, activator

## Abstract

*Mycoplasma gallisepticum* (MG) is recognized as a principal causative agent of avian chronic respiratory disease, inflicting substantial economic losses upon the poultry industry. However, the extensive use of conventional antibiotics has resulted in the emergence of drug resistance and various challenges in their clinical application. Consequently, there is an urgent need to identify effective therapeutic agents for the prevention and treatment of mycoplasma-induced respiratory disease in avian species. AMP-activated protein kinase (AMPK) holds significant importance as a regulator of cellular energy metabolism and possesses the capacity to exert an anti-inflammatory effect by virtue of its downstream protein, SIRT1. This pathway has shown promise in counteracting the inflammatory responses triggered by pathogenic infections, thus providing a novel target for studying infectious inflammation. Quercetin possesses anti-inflammatory activity and has garnered attention as a potential alternative to antibiotics. However, there exists a gap in knowledge concerning the impact of this activation on MG-induced inflammatory damage. To address this knowledge gap, we employed AlphaFold2 prediction, molecular docking, and kinetic simulation methods to perform a systematic analysis. As expected, we found that both quercetin and the AMPK activator AICAR activate the chicken AMPKγ1 subunit in a similar manner, which was further validated at the cellular level. Our project aims to unravel the underlying mechanisms of quercetin’s action as an agonist of AMPK against the inflammatory damage induced by MG infection. Accordingly, we evaluated the effects of quercetin on the prevention and treatment of air sac injury, lung morphology, immunohistochemistry, AMPK/SIRT1/NF-κB pathway activity, and inflammatory factors in MG-infected chickens. The results confirmed that quercetin effectively inhibits the secretion of pro-inflammatory cytokines such as IL-1β, TNF-α, and IL-6, leading to improved respiratory inflammation injury. Furthermore, quercetin was shown to enhance the levels of phosphorylated AMPK and SIRT1 while reducing the levels of phosphorylated P65 and pro-inflammatory factors. In conclusion, our study identifies the AMPK cascade signaling pathway as a novel cellular mediator responsible for quercetin’s ability to counter MG-induced inflammatory damage. This finding highlights the potential significance of this pathway as an important target for anti-inflammatory drug research in the context of avian respiratory diseases.

## 1. Introduction

*Mycoplasma gallisepticum* (MG) is an avian pathogen that is known to occur frequently in chickens and cause chronic respiratory diseases (CRDs) [1,2,3]. It has resulted in significant economic losses within the poultry breeding industry. Researchers reported that it is a highly infectious pathogen capable of causing persistent infection within the host and has the potential for horizontal and vertical transmission [4]. MG-induced CRDs inhibit weight gain, cause carcass condemnation in broilers, and have substantial negative effects on the laying rate in laying hens [5,6]. Prevention becomes difficult because MG can cause repeated infections. Up to now, the prevention and treatment of MG disease still rely on antibiotics, such as tiamulin, doxycycline, and fluoroquinolones, which can alleviate the threat posed by MG. However, the long-term and large-scale use of antibiotics has resulted in many problems, such as bacterial resistance and the food safety risks caused by drug residues. Hence, the primary focus of this study is to discover preventive and therapeutic approaches against MG infection that can serve as viable alternatives to antibiotics.

Quercetin is a flavonoid widely found in plants and fruits, 3,3′,4′,5,7-pentahydroxyflavone with three rings and five hydroxyl groups, which has anti-inflammatory, antioxidant, antiviral, anti-tumor, and immunomodulatory effects [7]. Quercetin has been demonstrated to possess potent anti-inflammatory properties, effectively inhibiting the expression of inflammatory cytokines and the activity of inflammatory response proteases both in vivo and in vitro, thus exhibiting strong anti-inflammatory effects. It is one of the ideal alternative antibiotics [8]. Quercetin has been shown to reduce the production of LPS-stimulated inflammatory cytokines TNF-α, IL-1β, and IL-6 in macrophages by inhibiting NF-κB [9,10,11]. Recently, it was found to inhibit the inflammatory response of carotid atherosclerosis induced by a high-fat diabetic diet in rats by regulating the AMPK/SIRT1/NF-κB pathway [12], and also reduced inflammation in obese mice via the AMPKα1/SIRT1 pathway [13]. However, studies on the anti-inflammatory effects of quercetin on the prevention and treatment of diseases related to livestock and poultry are rare, and most of them focus on the protein and lipid content of chicken.

5’-adenosine monophosphate (AMP)-activated protein kinase (AMPK) is a key regulator of cellular energy, and it maintains cellular energy homeostasis by coordinating many pathways [14]. Some evidence has indicated that AMPK relies on its downstream protein SIRT1 to inhibit inflammation in pathological conditions [15,16,17]. SIRT1 is a kind of NAD (+)-dependent deacetylase that acts as a downstream protein of the AMPK signaling pathway and exerts anti-inflammatory effects by inhibiting NF-κB [18,19,20]. Quercetin was shown to achieve anti-inflammatory effects by activating the AMPK/SIRT1 pathway [21], but it has not been reported whether quercetin can inhibit MG inflammatory damage by activating the AMPK pathway.

To clarify the correlation between quercetin and AMPK/SIRT1 activation, we docked small molecules of quercetin with the three-dimensional structure of mammalian AMPK. Using molecular docking analysis, the results showed that the binding patterns of quercetin, ZMP, and AMPK were similar. AICAR nucleoside monophosphate (ZMP), a phosphorylated product of AICAR inside cells, mimics the multiple effects of AMP on AMPK allosteric activation without changing nucleotide levels [22]. ZMP binds to the CBS1 or CBS3 binding sites of the AMPK-γ subunit to allosterically activate AMPK, and coincidentally, quercetin binds to AMPK residues with high similarity. Therefore, quercetin and AICAR are predicted to have similar effects on AMPK activation. Building upon the established anti-inflammatory effect of the quercetin-mediated AMPK/SIRT1 pathway, along with the molecular docking results from this investigation, our study aims to elucidate the correlation between the AMPK cascade signaling pathway and MG-induced inflammation. Furthermore, we seek to comprehensively understand the anti-inflammatory mechanism of quercetin-mediated AMPK activation in both in vitro and in vivo settings, utilizing insights gained from bioinformatics analysis. By achieving these research objectives, our study not only advances the current understanding of the molecular pathways involved in MG-induced inflammation but also presents a promising avenue for identifying and developing non-antibiotic-specific drugs to combat MG infection. This novel approach paves the way for exploring alternative treatment strategies and lays a solid foundation for further research and development of anti-inflammatory drugs that target the AMPK pathway. Ultimately, our findings offer valuable contributions to the search for effective therapeutic options against MG infection while reducing reliance on antibiotics, and provide a basis for the future advancement of anti-inflammatory interventions centered on AMPK.

## 2. Results

### 2.1. AMPK Model Building and Quality Assessment Visualization

**Figure 1:**
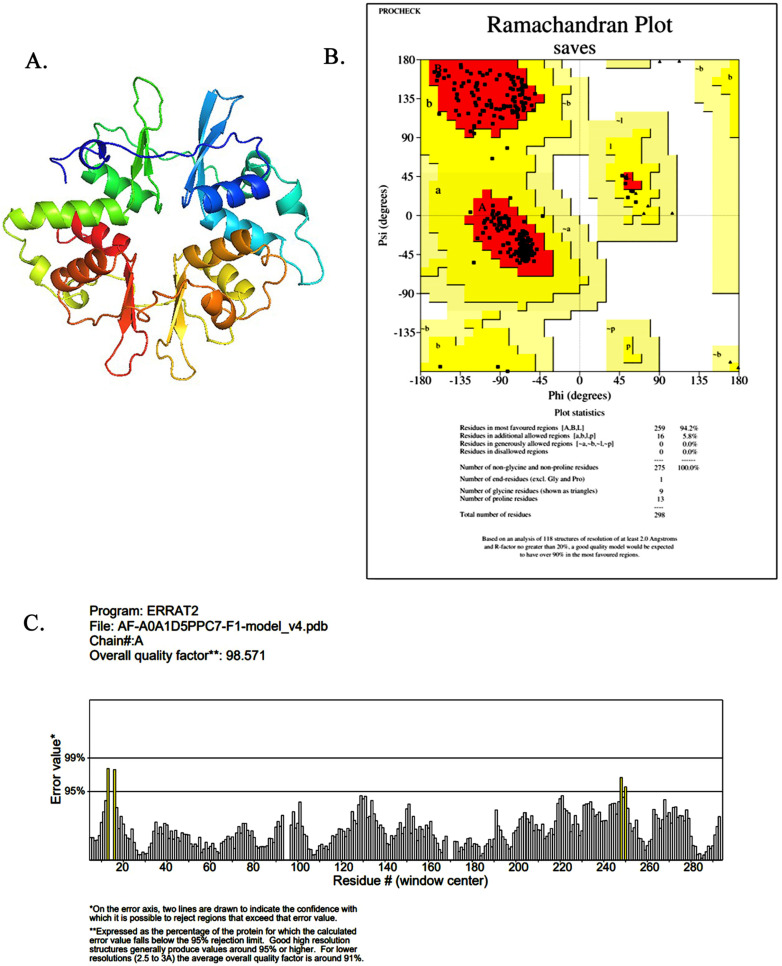
The predicted structure and evaluation of AMPKγ1 in chicken. (**A**) The chicken AMPKγ1 subunit. (**B**) Ramachandran plot of AMPKγ1 subunit model structure was verified using the PROCHECK program. The red areas indicate the most favorable areas for amino acid residues; the yellow areas indicate a large number of allowed areas; and the brown areas indicate additional permitted areas. The Ramachandran plot of the AMPKγ1 subunit contains 94.2% residues in the favorable and permissible regions, indicating that the protein has a good-quality model. (**C**) ERRAT evaluation of chicken AMPKγ1 subunit structural model.

In this study, the γ1-subunit of the chicken AMPK model was obtained using AlphaFold2 online prediction (Figure 1A). Subsequently, ERRAT and PROCHECK were used to check the reliability of the created protein model and the three-dimensional structure error [23]. As shown in Figure 1B, the results showed that 94.2% of the residues were located in the most favorable region of the plot, indicating that the model is valid. The structure’s reliability score for each residue was determined to be 98.571 (Figure 1C), signifying a high level of accuracy and confidence. This score validates the usability of the model for subsequent studies. The findings conclusively indicate that the Ch-AMPKγ1 structure is not only compatible but also of excellent quality, establishing its credibility and suitability for further investigation.

### 2.2. Molecular Docking Analysis

**Figure 2:**
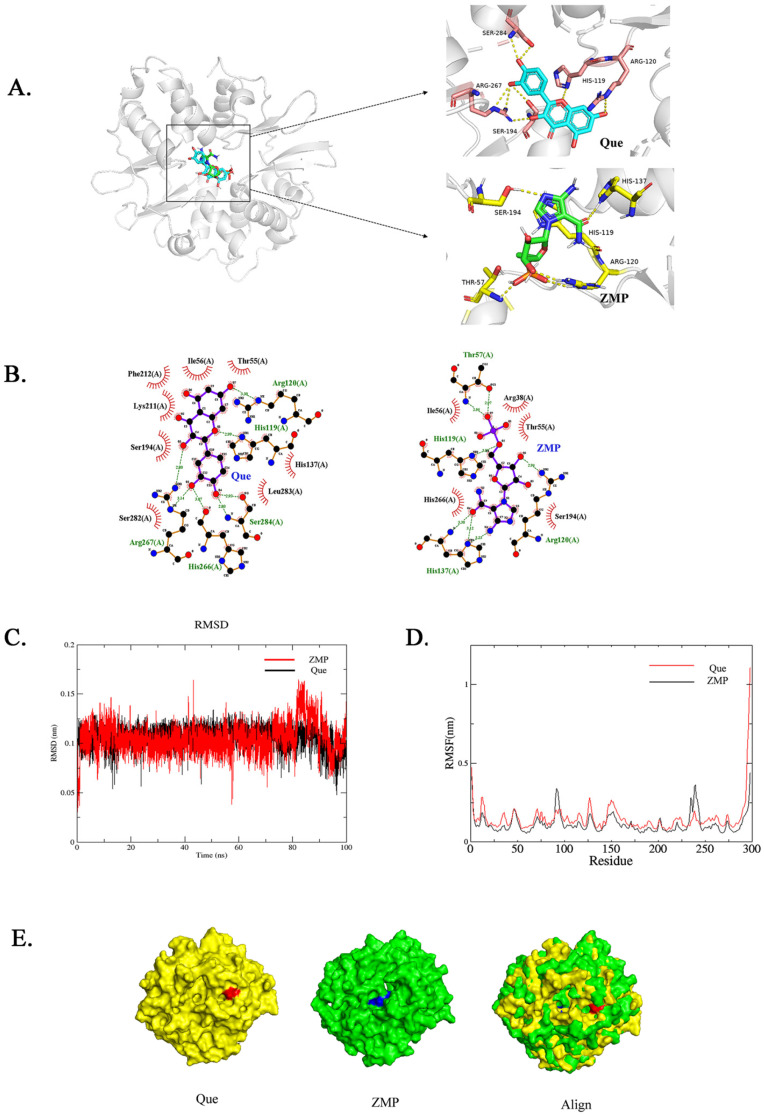
Molecular docking and the binding interaction of quercetin and AICAR to the γ-subunit of AMPK. (**A**) Docked pose of quercetin and AICAR with the AMPKγ1 subunit. (**B**) Hydrogen bonds and hydrophobic interactions between quercetin and the γ-subunit of AMPK were analyzed using the LigPlot program. The green dotted line indicates the hydrogen bonding between the residue and the ligand small molecule, and the red fan indicates the hydrophobic interaction between the residue and the ligand small molecule. (**C**) Plots of the root means squared deviation (RMSD) values during 100 ns in the simulation times corresponding to the molecular dynamics of all complexes under study. (**D**) Plots of the root mean square fluctuation (RMSF) values during 100 ns in the simulation times correspond to the molecular dynamics of all complexes under study. (**E**) PyMOL-visualized molecular dynamic simulations post ligand receptor binding. Both quercetin and ZMP (the active ingredient of AICAR after metabolism) bind to the Ch-AMPKr1 subunit.

To determine the potential interactions between quercetin and the Ch-AMPKγ1 subunits, AutoDock Vina was used for the molecular docking analysis while using ZMP as a positive control (Figure 2A). The intermolecular interaction between quercetin/ZMP and the Ch-AMPKγ1 subunit was analyzed. Figure 2B showed that hydrogen bonds were formed between quercetin and the Ch-AMPKγ1 subunit, including Arg120, His119, Ser284, His266, and Arg267, and the hydrogen bonds between ZMP and the Ch-AMPKγ1 subunit included Thr57, His119, His137, and Arg120. Two identical hydrogen bonds were formed between quercetin and the Ch-AMPKγ1 subunit, and between ZMP and the Ch-AMPKγ1 subunit. The following amino acid residues interacted with quercetin to form hydrophobic forces: Ile56, Thr55, Phe212, Lys211, Ser194, Ser282, Leu283, and His137. Hydrophobic forces formed between ZMP and Arg38, Ile56, Thr55, Ser194, and His266 of the Ch-AMPKγ1. The formation of hydrogen bonds and hydrophobic forces indicated that the binding mode between quercetin and the Ch-AMPKγ1 subunit was similar to that of ZMP.

To compare the experimental structural stability of the molecular dynamics simulations, we used two evaluation methods, including the RMSD and RSMF. The results are shown in Figure 2C: under the simulated conditions of the quercetin and Ch-AMPKγ1 subunits, the RMSD averaged 0.106 ± 0.013 nm, while the positive control of the ZMP small molecule averaged 0.105 ± 0.015 nm. Their fluctuation trends for the RMSD were in a reasonable range, and the RMSF curves of the two complex systems had similar fluctuation trends. Similarly, the RMSF fluctuations in the 25–275 residue region were relatively stable and small, indicating that the range was because the amino acids in the ligand molecules could be better combined (Figure 2D).

The binding situation between the ligand and the receptor after the molecular dynamics simulation was visualized using PyMOL (Figure 2E), and then the binding free energy of the complex was calculated using the MMPBSA algorithm. The binding energy of the quercetin-Ch-AMPKγ1 subunit complex was lower than that of the positive control ZMP. Indeed, in molecular interactions, a lower binding free energy corresponds to a stronger intermolecular interaction, leading to the formation of a more stable complex (Table 1).

The results of the study demonstrated a high affinity of quercetin towards the Ch-AMPKγ1 subunit. Based on these findings, it is proposed that quercetin may function as a positive modulator of AMPK, with its activation mode likely resembling that of AICAR.

### 2.3. Quercetin Mitigates MG Infection-Induced Inhibition of AMPK Activity in CP-II Cells

**Figure 3:**
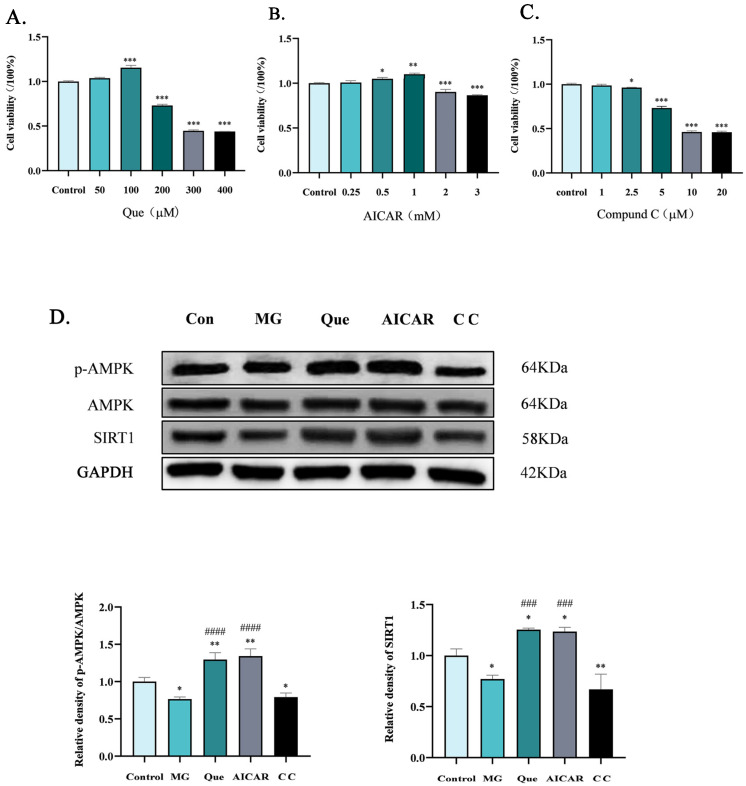
Quercetin alleviated inhibition of AMPK activity by MG infection in CP-II cells. (**A**) Effects of quercetin exposure on CP-II cell viability when using the CCK-8 assay (*n* = 5). Quercetin at the indicated concentrations at 24 h. (**B**) AICAR at the indicated concentrations at 24 h. (**C**) Compound C (C C) at the indicated times. (**D**) Effects of different drugs on the levels of p-AMPK/AMPK and SIRT1 after 24 h. Values are expressed as mean ± SD. *, *p* < 0.05 versus the results for the control; **, *p* < 0.01 versus the results for the control; ***, *p* < 0.001versus the results for the control; ###, *p* < 0.001 for comparison between treatments; ####, *p* < 0.0001 for comparison between treatments.

First, the effects of different concentrations of quercetin, AICAR, and Compound C in CP-II cells were investigated using a CCK-8 assay before investigating the effect of quercetin on AMPK activation in the CP-II cells. The results showed that concentrations of quercetin below 100 μM or AICAR below 10 mM had essentially little impact on cell viability, and even 100 μM concentrations of quercetin and 0.5–1 mM concentrations of AICAR showed the ability to facilitate cell survival (Figure 3A, *p* < 0.001). When 200 μM of quercetin and 2 mM AICAR concentrations showed the ability to inhibit cell survival (Figure 3B, *p* < 0.001). The concentration of Compound C exceeded 5 mM, and the cell viability was inhibited by more than 50% (Figure 3C, *p* < 0.001). Therefore, quercetin (100 μM), AICAR (1 mM), and Compound C (5 μM) were employed in the subsequent experiments.

To further determine the effect of quercetin on AMPK activation in chicken CP-II cells, AICAR was used as a positive control and Compound C was used as a negative control, respectively. The results showed that the p-AMPK levels were reduced in the MG and Compound C groups (Figure 3D, *p* < 0.05), while the AICAR and quercetin group increased significantly compared to the control group (*p* < 0.01). Compared to the MG infection group, the level of p-AMPK in the quercetin group and the AICAR group increased significantly (*p* < 0.0001).

### 2.4. Quercetin Reduces MG-Induced Release of Proinflammatory Cytokine

**Figure 4:**
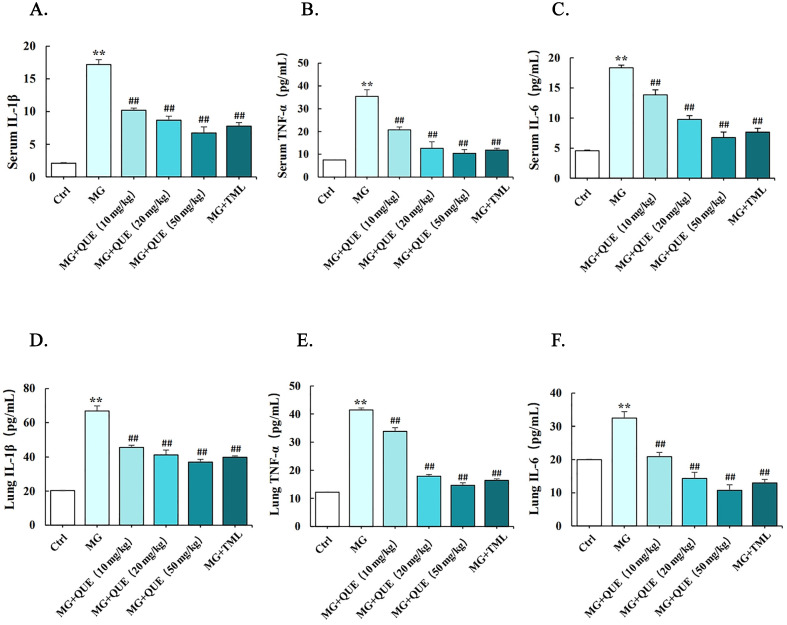
Quercetin regulates the level of inflammatory factors in MG-infected chickens. Quercetin regulates the level of inflammatory factors in MG-infected chickens. (**A**–**C**) Levels of serum IL-1β, TNF-α, and IL-6 in serum. (**D**–**F**) Levels of IL-6, TNF-α, and IL-1β in the lungs. Values are expressed as mean ± SD. ** *p* < 0.01 versus Ctrl; ## *p* < 0.01 versus MG.

To identify the anti-inflammatory effects of quercetin, ELISA was used to detect the release of marker inflammatory cytokines in serum and lung tissue due to MG infection. Compared to the control group, the marker inflammatory cytokines increased significantly in the serum and lungs of the MG group (*p* < 0.01). It has been found that the quercetin treatment group was consistent with the tiamulin group, and the serum and lung levels of IL-1β, IL-6, and TNF-α were significantly reduced compared with the MG group in a dose-dependent manner (Figure 4A–F, *p* < 0.01).

### 2.5. Quercetin Ameliorates Air Sac Diseases and Lung Damage following MG Infection

**Figure 5:**
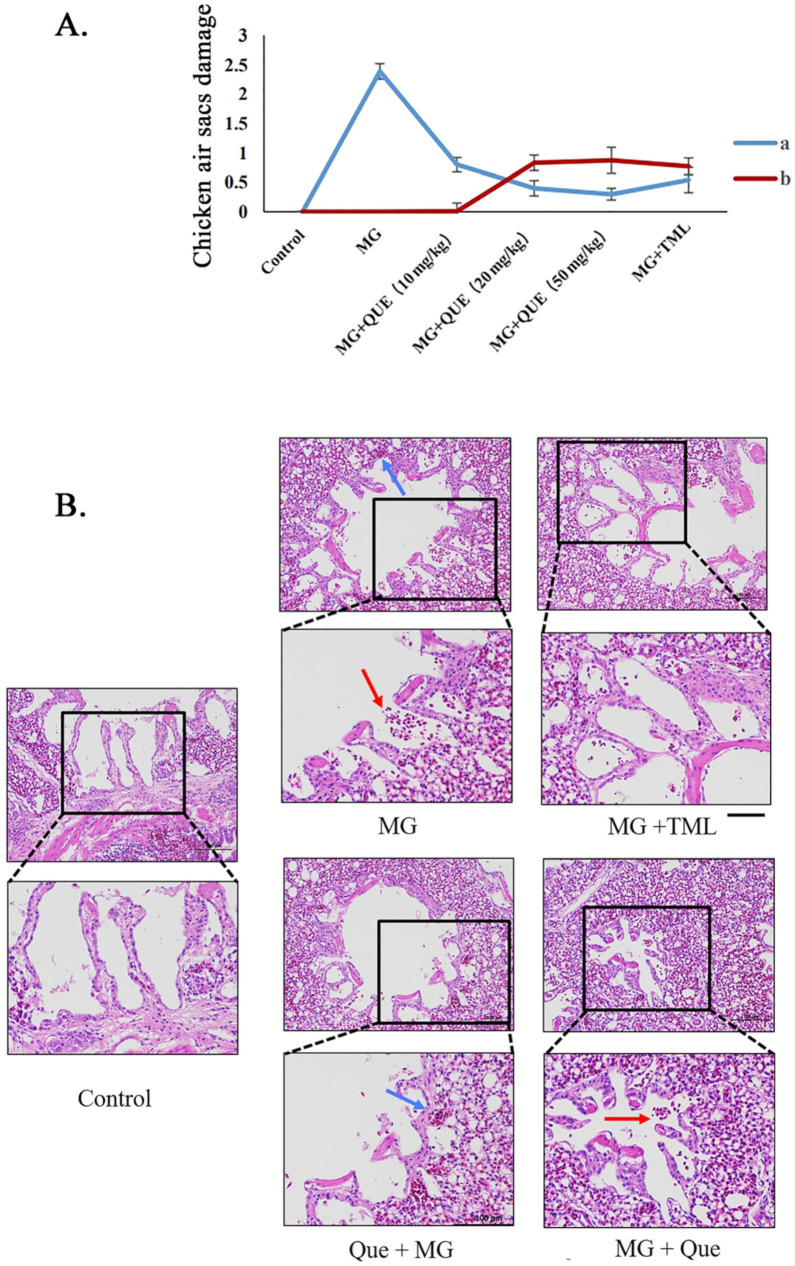
Quercetin alleviates air sac and lung damage in MG-infected chickens. (**A**) Evaluation of air sac pathologic damage in MG-infected chickens, where **a** represents the air sac damage score of different groups and **b** represents the airbag damage reduction rate. (**B**) HE staining to detect the pathological changes in lung inflammation caused by quercetin-inhibiting MG infection. The red arrows point to inflammatory cells, and the blue arrows point to red blood cells. Scale bar = 100 μm.

Seven samples were randomly selected from each group to analyze air sac injury and damage the reduction rate, respectively, aiming to observe whether quercetin could reverse the air sac and lung damage caused by MG infection. The airbag damage of the prevention group and the treatment group showed a decreasing trend compared with the other groups, indicating that quercetin could reduce the pathological damage of chicken air sacs caused by MG infection. The high-dose prevention group and high-dose treatment group experienced the best effect, and the lowest air sac damage score was 0.37 and 0.3, respectively (Figure 5A). The air sac damage reduction rate was 84.4% and 87.4%, respectively (Figure 5A). Therefore, a dosage of quercetin 50 mg/kg/d was selected in this study for subsequent experiments.

The effect of quercetin on MG-induced lung inflammation in chickens was further verified using HE staining (Figure 5B). The results showed that the ultrastructure of the lungs was not significant in the control group, and in the distribution of the two normal lung markings, there were clear lung fields, which was not substantive. The MG infection group showed hemorrhages, damaged alveoli, and infiltration of inflammatory cells into the lung interstitium. The alveolar and cell structure of the treated group and the positive control group were intact, and no obvious pathological phenomena were found. As anticipated, quercetin attenuates MG-induced inflammation in a dose-dependent manner in chicken lungs, with its therapeutic efficacy outweighing its preventive effect.

### 2.6. The Impact of Quercetin on the AMPK/SIRT1/NF-κB Pathway In Vivo and In Vitro under MG Infection

**Figure 6:**
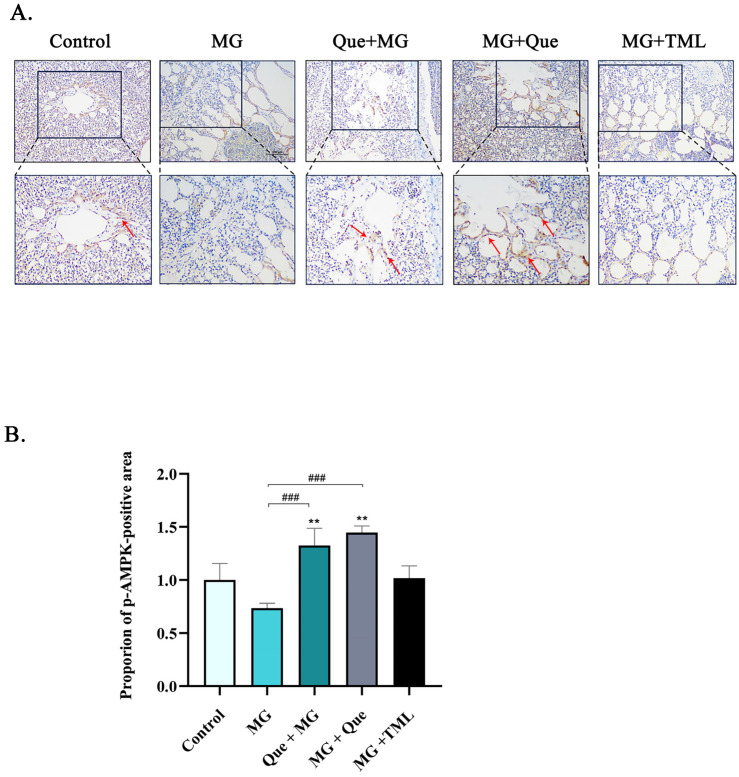
Effect of quercetin on the expression of MG-induced protein in CP-II cells. (**A**) The expression level of p-AMPK in chicken lungs was detected using immunohistochemistry. The brown color indicates the p-AMPK region. The red arrow points to the positive stained area. Scale bar = 100 μm. (**B**) Image-J software analyzed the proportion of positive area in immunohistochemical results (*n* = 3). Values are expressed as mean ± SD. ** *p* < 0.01 versus control; and ### *p* < 0.001 versus MG.

In this study, immunofluorescence (IHC) was performed to determine whether AMPK activation participates in quercetin attenuating MG-induced inflammation damage in vivo. IHC staining of chicken lung tissue sections showed that p-AMPK exhibited tissue-specific distribution in the chicken lungs. Compared to the control group, the pulmonary alveoli of the MG group were weakly positive in terms of the levels of p-AMPK protein in the lungs. While the quercetin prevention group and the treated group showed medium to strong positive staining, meanwhile, the positive control group and the control group showed a similar positive staining rate (Figure 6A). Furthermore, as shown in Figure 6B, we analyzed the area proportion of the positive immunohistochemical staining part.

**Figure 7:**
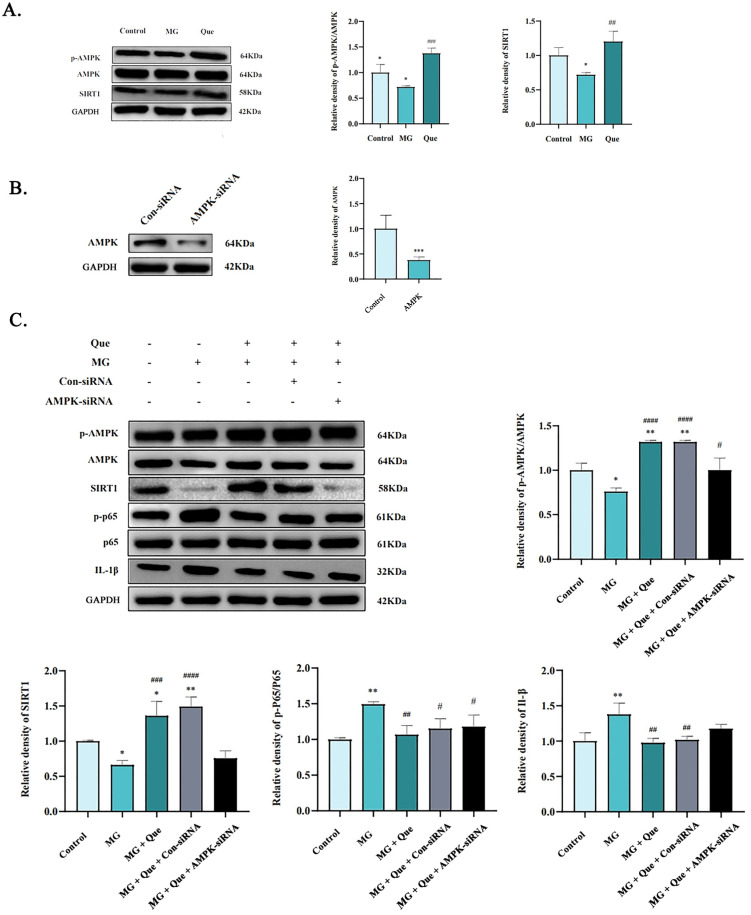
The expression of p-AMPK in the lungs of chickens infected with MG was detected using immunohistochemistry. (**A**) Western blot assays of the ratio of p-AMPK/AMPK, SIRT1 expression, and densitometric quantification. (**B**) CP-II cells transfected with AMPK siRNA/control siRNA, and AMPK expression significantly decreased after AMPK siRNA transfection into CP-II cells. *** *p* < 0.001 versus control siRNA group. (**C**) Relative expression of AMPK, the ratio of p-AMPK/AMPK, relative expression of SIRT1, p-P65/P65 and IL-1β expression, and densitometric quantification. Values are shown as mean ± SD, * *p* < 0.05 and ** *p* < 0.01 vs. control; # *p* < 0.05, ## *p* < 0.01, ### *p* < 0.001 and #### *p* < 0.0001 vs. MG.

To identify the potential mechanism by which quercetin alleviated MG-induced lung inflammatory damage in chickens, we evaluated the effect of quercetin on the activation of the AMPK/SIRT1 pathway in the lungs after MG infection. Western blot data showed that the p-AMPK levels in the MG-infected group were significantly lower than those in the control group (*p* < 0.05). Nevertheless, quercetin exhibited a notable increase in AMPK phosphorylation and effectively induced the expression of SIRT1 protein. Consequently, it significantly reversed the inhibition of AMPK activity caused by MG infection (Figure 7A, *p* < 0.01).

These results have confirmed that quercetin inhibits the lung inflammation induced by MG infection and reverses the inhibition of AMPK activity. Several pieces of evidence showed that SIRT1 was a downstream target of the AMPK signaling pathway and participated in anti-inflammation through the suppression of NF-κB p65 activation [18,19,20]. To further verify whether quercetin exerts anti-inflammatory effects in MG-infected CP-II cells via the AMPK/SIRT1/NF-κB pathway, we artificially reduced the expression of the AMPK gene in CP-II cells using AMPK-siRNA (Figure 7B, *p* < 0.001). An MG-induced inflammatory damage model continued to elucidate the anti-inflammatory effects of quercetin therapy. The results from Figure 7C showed that the ratio of p-AMPK/AMPK in the MG group was lower than in the control group (*p* < 0.05), indicating that MG inhibited the expression levels of p-AMPK and SIRT1. However, the ratio of AMPK/AMPK and the SIRT1 protein level increased significantly, while the ratio of p-p65/p65 and IL-1β reduced remarkably in the quercetin-treated CP-II cells compared to the cells exposed to MG only (*p* < 0.01). It is worth noting that the above trend caused by quercetin is reversed by siRNA-AMPK. Compared to the MG group, the quercetin-treated group significantly decreased the relative expression of p-NF-κB and IL-1β with the activation of the AMPK/SIRT1 pathway (Figure 7C, *p* < 0.05). The combined data in Figure 7 further confirmed quercetin’s anti-inflammatory effect by regulating the AMPK/SIRT1/NF-κB pathway.

## 3. Discussion

MG is widely recognized as the “source of all diseases” in the poultry industry. Once the host becomes infected, MG persists throughout its life. The disease is characterized by both vertical and horizontal transmission, making it a significant concern for poultry health and industry management [24]. MG infection cause chronic respiratory diseases in poultry, which severely affects the egg production rate, hatching rate, and poultry feed conversion rate [24,25,26]. After infection with MG, it is difficult to cure and eradicate. It is easy to cause serious secondary or mixed infections, resulting in a sharp increase in poultry mortality and bringing heavy economic losses to the poultry breeding industry. Therefore, effective prevention and control of MG is particularly important. Until now, tetracycline and macrolide antibiotics such as tiamulin have been commonly used in the clinical treatment of MG infection [27]. Although antibiotics treatment has a certain alleviating effect on MG infection, the large number of antibiotics in livestock and poultry farming and the irregular use of antibiotics result in a reduced sensitivity of strains to antibiotics and increased resistance. Equally, the frequent occurrence of major problems such as drug residues is of great concern to consumers, and, for these reasons, the difficulty of MG infection prevention and control is aggravated. Therefore, there is an urgent need to find non-antibiotic-specific anti-inflammatory drugs to prevent and treat MG infections.

In the study of natural products, quercetin has been found to have anti-inflammatory, antioxidant, antitumor, and antiviral properties, alleviate cardiovascular disease, regulate immunity, and perform other drug activities [28,29]. As an antitumor and antioxidant, it has achieved good efficacy. In recent studies, the anti-inflammatory effect of quercetin has garnered significant attention, emerging as a standout among natural products with its remarkable potency as an anti-inflammatory drug. Quercetin played an anti-inflammatory role both in vivo and in vitro by inhibiting the activity of inflammatory protease and the expression of inflammatory factors and is considered one of the ideal drugs to replace antibiotics [30]. However, in relevant research in the field of livestock and poultry, research on quercetin focuses only on the protein content and lipid content of poultry meat [31,32], and researches on the use of quercetin in the prevention and treatment of diseases related to livestock and poultry are rare.

As we all know, AMPK is a key regulatory molecule of intracellular energy metabolism [33]. It is a heterotrimer protein composed of α, β, and γ subunits, and it is widely divided into different tissues and organs of the body. It can be activated using upstream protein kinases (such as LKB1, etc.), substances that affect cell metabolism (such as ADP, AMP, etc.), and hormones and AMPK activators (AICAR, metformin, etc.), and improve the level of intracellular ATP by reducing energy-consuming anabolism and increasing productivity catabolism, thereby maintaining cellular energy balance [34,35]. Several studies have confirmed that the activation of AMPK can significantly reduce inflammatory damage and the expression of cellular inflammatory factors [36]. Furthermore, it has been confirmed that AMPK is significantly activated in studies of resistance to pathogen infection, LPS-induced lung damage, and other inflammatory reactions [37,38]. Many evidences verify the inhibitory effect of the AMPK/SIRT1/NF-κB pathway on inflammation [17,20]. The anti-inflammatory mechanism of this pathway may provide a new target for the prevention and treatment of infectious inflammatory diseases.

**Figure 8:**
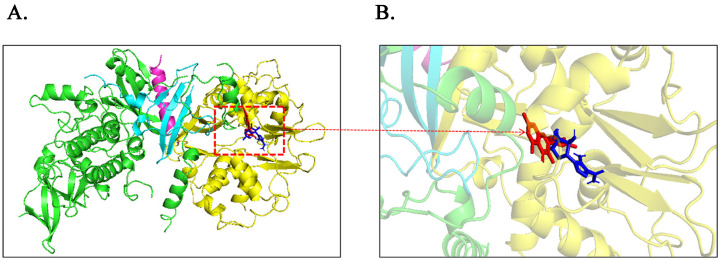
The active structure of mammalian AMPK (PDB ID: 4CFH) was docked with quercetin and ZMP, respectively, and stably bound to the AMPKγ subunit. (**A**) Green is the mammalian AMPKα subunit, blue is the β subunit, and yellow is the gamma subunit; (**B**) Quercetin was colored red, and ZMP was colored blue.

In the present study, we focused on the anti-inflammatory mechanism of quercetin. Before formal experiments, we found that quercetin binds stably to the mammalian AMPKγ subunit when using molecular docking, and the binding way is consistent with the binding way of the active ingredient ZMP in AICAR, an activator widely used in research of the AMPK mechanism (Figure 8). Therefore, it is speculated that quercetin may have the property of activating AMPK. This is complementary to the relevant research results that quercetin exerts anti-inflammatory effects by activating AMPK and the downstream protein molecule SIRT1 in LPS-induced macrophage inflammation, obesity-induced inflammation, diabetes-induced inflammation, and other inflammatory responses [39,40,41].

To this end, we systematically explored the correlation between quercetin and AMPK using bioinformatics methods. AlphaFold2 was used to predict and obtain the chicken AMPKγ1 subunit model online. ZMP (AMP analog, the 5-hydroxyl of AICAR is converted using adenosine kinase phosphorylation in vivo) was used as the positive control group. The results of the molecular docking showed that the quercetin and ZMP binding sites were similar to those of the chicken AMPKγ1 subunit, and both were bound near the active center of the domain (Figure 2A). The kinetic simulation results showed that the stable binding of quercetin to chicken AMPKγ1 had a mode of action similar to that of AMPK in activating AICAR (Figure 2E). It is suggested that the stable binding of quercetin and chicken AMPK may be a way to activate AMPK in chickens. In the present study, the AMPK activator AICAR and Compound C, a specific inhibitor of AMPK, were used in CP-II cells infected with MG. Our results reveal that MG infection inhibits AMPK activity (Figure 3D). This is consistent with the findings of decreased AMPK activity (p-AMPK level) in the lungs in a mouse model of Streptococcus pneumoniae infection [42] and decreased p-AMPKα levels in the optic reticulum of rats infected with Staphylococcus aureus [43]. That is, pathogen infection generally leads to a decrease in AMPK activity. In addition, quercetin was also verified to activate AMPK in chicken CP-II cells (Figure 3D). Subsequently, we specifically knocked down AMPK and confirmed that quercetin had the effect of reversing the decrease in AMPK activity caused by MG infection, as shown in Figure 6C.

Based on the above results, we carried out a study on the prevention and treatment of quercetin on respiratory tract inflammation caused by MG infection in chickens and established a positive tiamulin control group. Our data revealed that quercetin could reduce inflammatory cell infiltration in lung tissue, promoted alveolar structure integrity and ameliorated lung inflammatory damage (Figure 4), inhibited the expression and secretion of specific inflammatory factors (IL-1β, TNF-α and IL-6) caused by MG infection (Figure 4), and was accompanied by an elevated level of p-AMPK in the lung tissue (Figure 6). Our results revealed the inhibitory effect of quercetin on MG-induced inflammatory damage and speculated that its anti-inflammatory effect was related to the promotion of AMPK phosphorylation. Furthermore, by comparing the anti-inflammatory efficacy of quercetin in preventing and treating MG infection, it was unexpectedly found that the therapeutic effect of quercetin was better than that of preventing MG infection.

**Figure 9:**
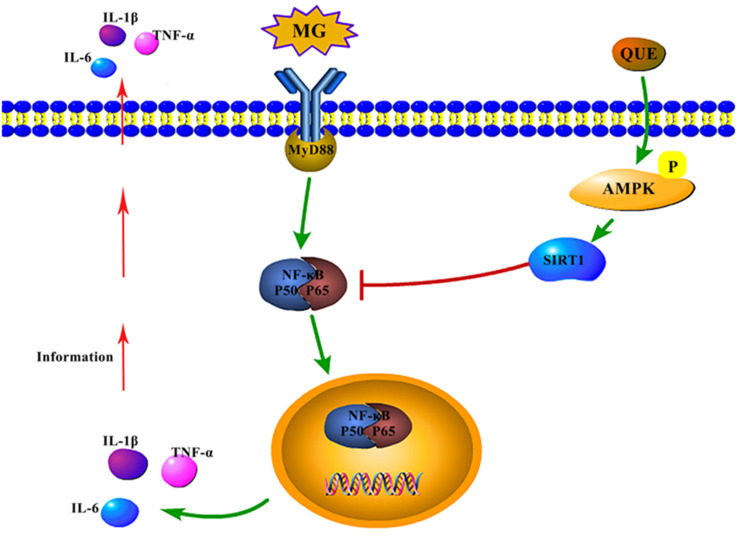
The mechanism of quercetin mediating the AMPK/SIRT1/NF-κB pathway to reduce MG-induced inflammatory damage.

Some evidence supports the AMPK/SIRT1/NF-κB pathway inhibiting inflammation [17,20,44], but the inflammatory response induced by MG infection has not been reported. In this study, MG infection was found to cause a decrease in the activity of the AMPK/SRIT1 pathway in the lung, which in turn enhanced the transcriptional activation of NF-κB, leading to an increase in the pro-inflammatory factor IL-1β (Figure 7A). This result is similar to what Wu et al. found in lipopolysaccharide (LPS)-induced mouse macrophages. The continuous activation of AMPKα by GSK6 to inhibit the activity of NF-κB is consistent with the expression of the pro-inflammatory factor TNF-α [45]. However, it is not clear whether the anti-inflammatory effect of the AMPK activator can be applied to the prevention and treatment of MG infectious diseases. To this end, we further elucidated the molecular mechanism of quercetin in reducing MG-induced inflammatory damage. We specifically knocked down the AMPK gene and established the MG infection model of chicken CP-II cells. After quercetin treatment, we detected changes in the level of pathway proteins, and we were fortunate to find that quercetin significantly activated the AMPK/SIRT1 pathway in MG-induced CP-II cells, thus inhibiting the transcriptional activity of NF-κB and IL-1β, reducing the expression of inflammatory factors (Figure 7C). From what has been discussed above, quercetin mediates the AMPK/SIRT1/NF-κB signaling pathway to inhibit the MG-induced inflammatory response (Figure 9). In particular, the findings of our partners also confirm quercetin’s ability to resist MG-induced inflammation, an anti-inflammatory mechanism that works by inhibiting the activity of the TLR2/myD88/NF-kB pathway [46]. Quercetin is suggested to achieve multi-target anti-inflammatory pharmacological effects.

However, it is important to note that the main barrier to quercetin utilization is bioavailability. Due to its low water solubility and stability, its bioavailability is relatively low. In order to solve the problem of a low quercetin absorption rate, relevant studies have found that quercetin can be significantly improved in its absorption rate and drug efficacy (such as better strengthening the immune and antiviral effects) by being combined with vitamin C and bromelain [47,48]. Quercetin-DHA ester-based pectin conjugates have increased its bioavailability and endowed quercetin with better antioxidant properties [49]. Therefore, improving quercetin’s bioavailability to improve quercetin’s anti-inflammatory effect will become one of our further research aims.

## 4. Materials and Methods

### 4.1. Construction of the 3D Structure of AMPK-γ Proteins

In this study, the chicken AMPKγ1(Ch-AMPKγ1) subunit sequence was obtained from the UniProt website (https://www.uniprot.org/, accessed on 20 October 2022), and the complete sequence of the Ch-AMPKγ1 subunit (A0A1D5PPC7 A0A1D5PPC7_CHICK) was obtained. Therefore, the Ch-AMPKγ1 subunit sequence was selected as the AlphaFold2 prediction template. The AlphaFold2 online server was used to input the primary amino acid sequence and homologous arrangement sequence to directly predict the 3D coordinates of all the heavy atoms of a given protein and verify the results [50].

### 4.2. Molecular Docking

The 3D structures of quercetin (PubChem CID5280343) and ZMP (PubChem CID 90471726) were obtained from PubChem. The download format was SDF and was converted into PDBQT format using OpenBabel. Meanwhile, the Ch-AMPKγ1 subunit protein receptor was pretreated with AutoDockTools v1 and saved in PDBQT format via AlphaFold2 prediction.

The docking experiments were performed using the AutoDock Vina module (Molecular Graphics Lab, The Scripps Research Institute). The LigPlot+v.2.2.5 and PyMOL-2.5.4-Windows-x86_64 software were used to analyze the interaction mode of the Ch-AMPKγ1 subunit receptor with the small molecules of the quercetin and ZMP ligand, and an interaction diagram between proteins and small molecules was drawn.

### 4.3. Molecular Dynamic Simulations

Molecular dynamic simulations were performed as previously described [51]. After exact docking, topological structures and coordinate files that simulated the protein-ligand complexes were created using Gromacs (version 2021.2). After constructing the structure of Ch-AMPKγ1, molecular dynamics (MD) simulation with quercetin and ZMP (the 5-hydroxyl group of AICAR, which is phosphorylated with adenosine kinase in analog AMP and activated AMPK), respectively. The interaction between quercetin–Ch-AMPKγ1 and ZMP–Ch-AMPKγ1 was compared and analyzed. The root means square deviation (RMSD) and root mean square fluctuation (RMSF) were used to predict the stability and binding energy of the simulation system. Therefore, the MM-PBSA calculation was used to calculate the binding free energy between ligands and proteins.

### 4.4. Mycoplasma Strains and Culture Conditions

The MG-R_low_ strain was provided by Northeast Agricultural University (Harbin, China). The MG was cultured at 37 °C in a modified Hayflicks medium supplemented with 20% FBS, 10% freshly prepared yeast extract, 0.05% penicillins, 0.05% thallium acetate, and 0.1% nicotinamide adenine dinucleotide (NAD). According to previous culture methods, MG was used for infection in the middle of the index [52], as shown by the color change in the red phenol dye from red to orange. The density of the MG in the culture medium was adjusted to 1 × 10^9^ CCU/mL (color change unit per milliliter) for subsequent experiments.

### 4.5. Cell Culture and Treatment

After 14 days of incubation with SPF chicken embryos, the lung tissue was cut into small pieces. Then, digest the small pieces in a 37 °C incubator using conventional methods for 15 to 20 min. The cell suspension was filtered through a 200-mesh sieve, and adhered to the conventional culture medium for 1 h. Then, the supernatant of the unconnected cells was filtered and collected three times using a 400-mesh sieve, centrifuged at 1200 r/min for 5 min, and resuspended in fresh Dulbecco’s modified Eagle medium (DMEM). Finally, the cells were counted and mixed with 20% FBS to adjust the concentration to 1.0 × 10^6^/mL, inoculated on a 150-mm^2^ sterile culture plate, and incubated at 39 °C for 18 h. The attached cells were CP-II cells. Subsequently, a routine culture was performed until the cells were fully grown for subsequent experiments.

To determine the concentrations of QUE, AICAR, and Compound C, CCK-8 assay (Shanghai Yuanye Biotechnology Co., Shanghai, China) was employed. After reaching confluence, the CP-II cells were treated for 24 h with different concentrations of QUE (10 μM, 20 μM, 40 μM, 80 μM, and 160 μM), AICAR (25 μM, 50 μM, 100 μM, 200 μM, and 400 μM) and Compound C (1μM, 2.5μM, 5 μM, 10 μM, and 20 μM), respectively. Finally, the CCK-8 reagent was added and the optical density (OD) was measured at 450 nm. Therefore, AICAR is a widely used AMPK activator, and it was used as a positive control drug for MG in this study; Compound C (CC) is a common AMPK inhibitor and was used as a negative control drug for MG in this study.

### 4.6. RNAi Transfection

The small interfering RNA targeting AMPK was obtained from GenePharma Co., Ltd. (Shanghai, China). The AMPK-siRNA transfection experiment was carried out as previously described with some modifications [52]. Briefly, the pretreated CP-II cells were transfected into cells with 75 pmoL AMPK/control siRNA with a Lipofectamine 2000™ reagent according to the instructions.

### 4.7. Experimental Chickens and Treatments

**Figure 10:**
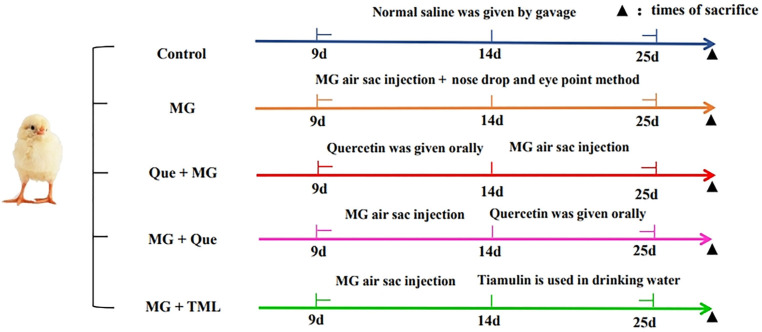
Detailed grouping and treatment of experimental animals.

The Great Wall Chicken Farm located in Anshan (Liaoning, China) provided one-day-old healthy laying hens that were acclimatized for 9 days before the experiment. The chickens were randomly divided into five groups, including the control group, the model group (MG infection group), the MG infection prevention group with quercetin (50 mg/kg/d), the model group treated with quercetin (50 mg/kg/d), and the model group treated with tiamulin (TML 25 mg/kg/d). Ten chickens were randomly assigned to each group in three replicates. The chickens were then challenged with 0.2 mL of the MG strain R_low_ (mid-log phase) at a concentration of 1 × 10^9^ CCU/mL in the left air sacs in the thoracic region [53]. Three days later, nasal and eye drops were administered at a concentration of 1 × 10^9^ CCU/mL MG strain R_low_ (mid-log phase) to assist with infection. Detailed grouping is shown in Figure 10. All of the experimental protocols were approved by the Liaoning University and Animal Use Committee prior to initiation of the study in accordance with the Guidelines on Welfare and Ethical Review for Laboratory Animals (GB/T 35892-2018, National Standards of the People’s Republic of China).

### 4.8. ELISA Assay

The serum and lung tissues of each group were collected and the supernatant was used for ELISA assay. The levels of the pro-inflammatory cytokines IL-1β, TNF-α, and IL-6 after treatment for MG-infected chickens with quercetin were measured using commercial ELISA kits from R&D Systems. The procedures were performed according to the manufacturer’s instructions using a microplate reader (Bio-Rad, Hercules, CA, USA).

### 4.9. Western Blot

The tissues and CP-II cells were lysed with a protein extraction reagent containing protease inhibitors and then centrifuged to obtain the proteins. The whole tissue lysate (60 μg) or cell lysate (20 μg) was separated using 8~12% SDS-PAGE, and then the proteins were blotted onto the PVDF membrane. Blocked with 5% (*w*/*v*) non-fat milk, the membrane was incubated at 4 °C overnight using primary antibodies corresponding to the proteins p-AMPK (Thr172), AMPK, SIRT1, p-p65 (S536), and IL-1, and further immersed in the corresponding HRP-conjugated secondary antibody for 1.5 h at room temperature (RT). All blots were detected using the enhanced chemiluminescence (ECL) method (Advansta, Hercules, CA, USA). The scanned images were quantified using Image J (version 1.47).

### 4.10. HE Staining

Histopathological examination was performed as previously reported [54]. Briefly, the lung samples in each group were fixed in 10% formalin overnight, dehydrated in a series of alcohol solutions, and embedded in wax. After embedding, the samples were cut into sects (5 μm).A hematoxylin and eosin (HE) staining solution was used for staining and observed under a light microscope (Nikon E100, 40× magnification, Tokyo, Japan).

### 4.11. Immunohistochemistry

The immunohistochemistry procedure was carried out as previously described with some modifications [53]. In brief, the endogenous peroxidase activity was quenched and incubated with the p-AMPK antibody (Bioss, Beijing, China) at 4 °C overnight, and the sections were washed three times with PBS. Then, an anti-mouse or anti-rabbit EnVision horseradish peroxide-labeled polymer (DAKO) was administered at 37 °C for 60 min. Finally, the reaction was observed with 0.05% 3-3’-diaminobenzidine and 0.03% hydrogen peroxide in a Tris hydrochloric acid buffer and then re-trained with Mayer’s hematoxylin.

### 4.12. Ethical Statement

The Institutional Animal Care and Use Committee of Liaoning University approved all procedures used in this study.

### 4.13. Statistical Analysis

All data were expressed as the mean ± SD. Statistical significance was determined using Student’s *t*-test or a one-way analysis of variance (ANOVA) in the case of comparisons among more than two groups following Dunnett’s T3 test, and the means had a normal distribution. A value of *p* < 0.05 was considered statistically significant. All graphs were made using GraphPad Prism (Version 8.0.1).

## 5. Conclusions

In summary, this study establishes that quercetin, as an activator of AMPK, effectively mitigates inflammation and ameliorates air sacs and alveolar lesions in chickens infected with MG by modulating the AMPK/SIRT1/NF-κB pathway. These findings present a novel approach to identifying and developing non-antibiotic-specific drugs to combat MG infection. Moreover, the study lays a solid foundation for further research and development of anti-inflammatory drugs that target the AMPK pathway, offering promising prospects for controlling the inflammatory damage caused by MG and contributing to the search for alternative treatment strategies in poultry health management.

## Figures and Tables

**Table 1:** MMPBSA calculating the binding free energy.

Complexes (Kcal/mol)	ΔG_total_	ΔG_vdw_	ΔG_ele_	ΔG_gas_	ΔG_polar_	ΔG_nonpolar_	ΔG_solv_
Que-AMPK	−53.216	−136.123	−63.101	199.224	166.718	−20.710	146.008
ZMP-AMPK	−4.545	−128.428	−185.410	−313.838	331.144	−21.852	309.292

## Data Availability

Not applicable.
